# Role of GDF15 in methylseleninic acid-mediated inhibition of cell proliferation and induction of apoptosis in prostate cancer cells

**DOI:** 10.1371/journal.pone.0222812

**Published:** 2019-09-20

**Authors:** Wenbo Zhang, Cheng Hu, Xiaojie Wang, Shanshan Bai, Subing Cao, Margaret Kobelski, James R. Lambert, Jingkai Gu, Yang Zhan

**Affiliations:** 1 National Engineering Laboratory for AIDS Vaccine, School of Life Sciences, Jilin University, Changchun, Jilin, China; 2 Department of Structural and Cellular Biology, Tulane Cancer Center, School of Medicine, Tulane University, New Orleans, Louisiana, United States of America; 3 Department of Pathology, University of Colorado School of Medicine, Aurora, Colorado, United States of America; Southern Illinois University School of Medicine, UNITED STATES

## Abstract

The growth inhibitory efficacy of methylseleninic acid (MSA) in prostate cancer cells has been documented extensively. However, our understanding of the immediate targets that are key to the growth inhibitory effects of MSA remains limited. Here, using multiple preclinical prostate cancer models, we demonstrated *in vitro* and *in vivo* that GDF15 is a most highly induced, immediate target of MSA. We further showed that knockdown of GDF15 mitigates MSA inhibition of cell proliferation and induction of apoptosis. Analysis of gene expression data from over 1000 primary and 200 metastatic prostate cancer samples revealed that GDF15 expression is decreased in metastatic prostate cancers compared to primary tumors and that lower GDF15 levels in primary tumors are associated with higher Gleason scores and shorter survival of the patients. Additionally, pathways that are negatively correlated with GDF15 levels in clinical samples are also negatively correlated with MSA treatment in cultured cells. Since most, if not all, of these pathways have been implicated in prostate cancer progression, suppressing their activities by inducing GDF15 is consistent with the anticancer effects of MSA in prostate cancer. Overall, this study provides support for GDF15 as an immediate target of MSA in prostate cancer cells.

## Introduction

Prostate cancer is the most common non-skin cancer and the second leading cause of cancer death in American men. Surgery and radiation therapy are effective regimens for localized prostate cancer, but there is no cure for metastatic disease, for which androgen deprivation therapy is the first-line therapy. While androgen deprivation therapy is effective initially, progression to castration-resistant prostate cancer (CRPC) is almost inevitable [1]. Docetaxel-based chemotherapy is a standard of care for patients with metastatic CRPC [2]. However, about half of the patients do not respond to the treatment, and those who do respond become refractory within one year [2]. Several new therapies have been recently developed and approved for treating docetaxel-resistant metastatic CRPC, such as the new taxane cabazitaxel [3], the CYP17A1 inhibitor abiraterone that suppresses androgen biosynthesis [4], and the potent AR antagonist enzalutamide [5]. Nonetheless, the survival benefits remain modest (<5 months), and resistance develops in essentially all patients [3–5]. Thus, developing effective therapeutic modality for prostate cancer remains an urgent task.

Methylseleninic acid (MSA) and methylselenocysteine (MSC) are two methyl-selenium compounds that have been demonstrated by many *in vitro* and *in vivo* studies to have potent anticancer activities against prostate cancer [6–24], and the safety profile of MSC has been established in humans [25]. It is important to appreciate that the anticancer efficacy of selenium compounds depends on the form and dosage administered [13, 16, 26, 27]. For example, selenomethionine, the first-generation selenium compound that was used in the Selenium and Vitamin E Chemoprevention Trial and showed no protection against prostate cancer [28], has distinct biological and pharmacological properties from MSA and MSC [13, 16, 26, 27, 29]. Unlike MSA and MSC, selenomethionine is ineffective in suppressing the growth of prostate tumors in animal studies [13, 16]. This could be attributed to non-specific incorporation of selenomethionine into proteins in place of methionine, limiting its further metabolism [27]. In contrast, as monomethylated forms of selenium, MSA and MSC can be easily metabolized to the active anticancer metabolite methylselenol [27]. With regard to selenium dosage, most of the preclinical studies that showed a positive association between selenium administration and tumor inhibition were conducted using pharmacological doses of selenium, not the nutritional dose that was used in the Selenium and Vitamin E Chemoprevention Trial. Therefore, the use of potent selenium compounds, such as MSA and MSC, at pharmacological doses is essential for further developing selenium compounds for prostate cancer intervention.

The metabolism of MSC to the active metabolite methylselenol requires the activity of β-lyase, which is mainly expressed in the liver and kidney [27]. In contrast, MSA, as an oxidized form of methylselenol, is readily reduced to methylselenol through a non-enzymatic reaction in cells [26]. Due to the fact that prostate epithelial cells express a low level of β-lyase, MSA is 10 times more potent than MSC in affecting biological processes *in vitro*, and it can inhibit the growth of prostate cancer cells at *in-vivo*-relevant concentrations (2–10 μM) [8, 11, 26]. MSA also has excellent anticancer activity in animals [13, 16–18, 20].

In order to gain insights into the mechanisms underlying the anticancer activity of monomethylated selenium compounds in prostate cancer, microarray studies have been conducted previously to profile MSA-induced gene expression changes in PC-3 and LNCaP human prostate cancer cells [8, 21]. In the present study, we reanalyzed these microarray datasets to compile the list of most highly modulated genes in both cell models, and the analysis and downstream studies led to the identification of growth differentiation factor 15 (GDF15) as a rapidly induced target of MSA. GDF15 is a divergent member of the transforming growth factor-β (TGF-β) superfamily, and it is also known as macrophage inhibitory cytokine-1, prostate differentiation factor, prostate-derived factor, non-steroidal anti-inflammatory drug-activated gene-1, placental bone morphogenic protein, and placental TGF-β [30]. Similar to the role of TGF-β in cancer development and progression, both tumor-suppressing and tumor-promoting functions of GDF15 have been reported in different cancer types, including prostate cancer [30]. In the present study, we analyzed several publicly available clinical prostate cancer datasets encompassing over 1000 primary prostate cancer samples and over 200 metastatic CRPC samples to assess the role of GDF15 in prostate cancer progression, and we characterized the functional significance of GDF15 induction in mediating the effects of MSA in inhibiting cell proliferation and inducing apoptosis in prostate cancer cells.

## Materials and methods

### Cell lines and reagents

LNCaP and PC-3 cells were obtained from ATCC. LAPC4 cells [31] were provided by Dr. Charles Sawyers, then at the University of California, Los Angeles. All cell lines are cultured in RPMI1640 supplemented with penicillin, streptomycin, and 10% fetal bovine serum. Cells used in all experiments were within 3 months of resuscitation of frozen cell stocks established within 3 passages after receipt of the cells. Cell authentication was performed at the Genetica DNA Laboratories, and cells were regularly evaluated for mycoplasma contamination. MSA and MSC were purchased from PharmaSe.

### Microarray, RNA-seq, and proteomics data analyses

The microarray profiles of MSA-modulated genes in PC-3 and LNCaP cells were from previously published studies [8, 21]. The MSA-PC-3 dataset was generated using the Affymetrix U95A chip [8], and the MSA-LNCaP dataset was generated using a 3K custom cDNA microarray containing the genes that are modulated by MSA in the MSA-PC-3 dataset [21]. The data analysis was conducted following the previous publications [8, 21].

The TCGA RNA-seq data of 500 primary prostate cancer samples were downloaded from Genomic Data Commons. The RNA-seq data from 3 cohorts of a total of 159 metastatic CRPC samples were downloaded from dbGaP, including 51 samples from the “Stand Up To Cancer East Coast Prostate Cancer Research Group” (SU2C) project (dbGaP accession pht004946.v1.p1) [32], 74 samples from the Prostate Cancer Medically Optimized Genome-Enhanced Therapy (PROMOTE) study (dbGaP accession phs001141.v1.p1) [33], and 34 samples from the Beltran study (dbGaP accession phs000909.v1.p1) [34]. RNA-seq gene expression analysis was performed using RSEM [35]. The transcripts per million (TPM) values from the RSEM output were used for downstream analysis.

The Grasso [36], Taylor [37], and Erho [38] microarray datasets were downloaded from Gene Expression Omnibus with accession numbers GSE35988, GSE21034, and GSE46691, respectively. The data were normalized using the Single Channel Array Normalization algorithm [39].

Gene Set Enrichment Analysis (GSEA) was performed with 1000 permutations. For analysis of the MSA-PC-3 microarray dataset, the genes were ranked using the Manhattan matrix with a continuous phenotype profile that shows steadily increasing gene expression across the time series. For analysis of the compiled RNA-seq data of 500 primary prostate tumors and the 159 metastatic CRPC samples, the genes were ranked using the Manhattan matrix with continuous phenotype labels for GDF15. Both datasets were run against the hallmark gene sets in the Molecular Signatures Database.

The Excel file containing processed protein expression data from the Latonen proteomics study [40] was downloaded from the Nature Communications website.

### Quantitative Reverse Transcription-PCR (qRT-PCR)

qRT-PCR was performed using the TaqMan method. The TaqMan PCR primers and probes for GDF15 and β-actin were from Applied Biosystems. The qRT-PCR analysis was performed at least three times in triplicate, and GDF15 levels were normalized by β-actin levels.

### Western blot analysis

Western blot analysis was performed using a standard protocol. After blocking in blocking buffer, the membrane was incubated with a primary antibody overnight at 4°C, followed by incubation with a fluorescent-labeled secondary antibody for 1 hour at room temperature. Membranes were scanned and analyzed using an Odyssey^®^ Infrared scanner (LI-COR Bioscience). The following antibodies were used: anti-glyceraldehyde-3-phosphate dehydrogenase (GAPDH, Millipore) and anti-GDF15 (US Biological). The Western blot analysis was done at least three times, and GDF15 levels were normalized by GAPDH levels.

### 22Rv1 xenograft tumor model

The 22Rv1 xenograft tumors were from a previous study [20]. The mixture of Ketamine (100 mg/kg) and Xylazine (10 mg/kg) was intraperitoneally injected for anesthesia before tumor cell inoculation. The mice were inspected daily to determine any untoward effect and avoid any unnecessary suffering. Daily administration of MSA or MSC at 3 mg selenium/kg/day by an oral route was started when the tumors reached ~100 mm^3^. On Day 15, mice were euthanized by CO2 asphyxiation and tumors excised for molecular analysis. All animal procedures were approved by Tulane University Institutional Animal Care and Use Committee.

### shGDF15 transfection

The shGDF15 construct was generated as previously described [41]. LNCaP cells were transfected with the shGDF15 or the scrambled control (shCtrl) construct using the Lipofectamine 2000 and Plus reagent (Invitrogen) per instruction of the manufacturer. The cells were treated with MSA at 24 h after transfection. BrdU ELISA, Cell Death ELISA, and Western blot analysis were conducted at 24 h post-MSA treatment.

### Cell proliferation assay

Proliferation was measured by using the BrdU Cell Proliferation ELISA kit (Roche) per instruction of the manufacturer with minor modifications. Briefly, after labeling the cells with BrdU for 2 h, the WST-1 reagent (Roche), which quantitatively monitors metabolic activity of the cells, was added to the wells to a final concentration of 10%. The cells were incubated for an additional 2 h. The amount of formazan converted from WST-1 by the metabolically active cells was quantitated at 450 nm. After removing the medium, the cells were fixed and the DNA denatured for the incorporated BrdU to bind to a peroxidase-conjugated anti-BrdU antibody. The immune complexes were detected by the subsequent substrate reaction, and the reaction product was quantified by absorbance at 370 nm (reference wavelength at 492 nm). Culture medium without cells and cells incubated with the anti-BrdU-peroxidase antibody in the absence of BrdU were used as controls for nonspecific binding. The BrdU ELISA result was normalized by the WST-1 reading, which correlates directly with cell number. The experiment was performed three times in triplicate.

### Apoptosis detection

Detached cells were precipitated by centrifugation and pooled with attached cells. Cytoplasmic histone-associated DNA fragments were quantified by using the Cell Death Detection ELISA^PLUS^ Kit (Roche) per manufacturer’s protocol. The absorbance was measured at 405 nm (reference wavelength at 492 nm). The experiment was performed three times in triplicate.

## Results

### GDF15 is one of the most highly induced targets of MSA

**Fig 1:**
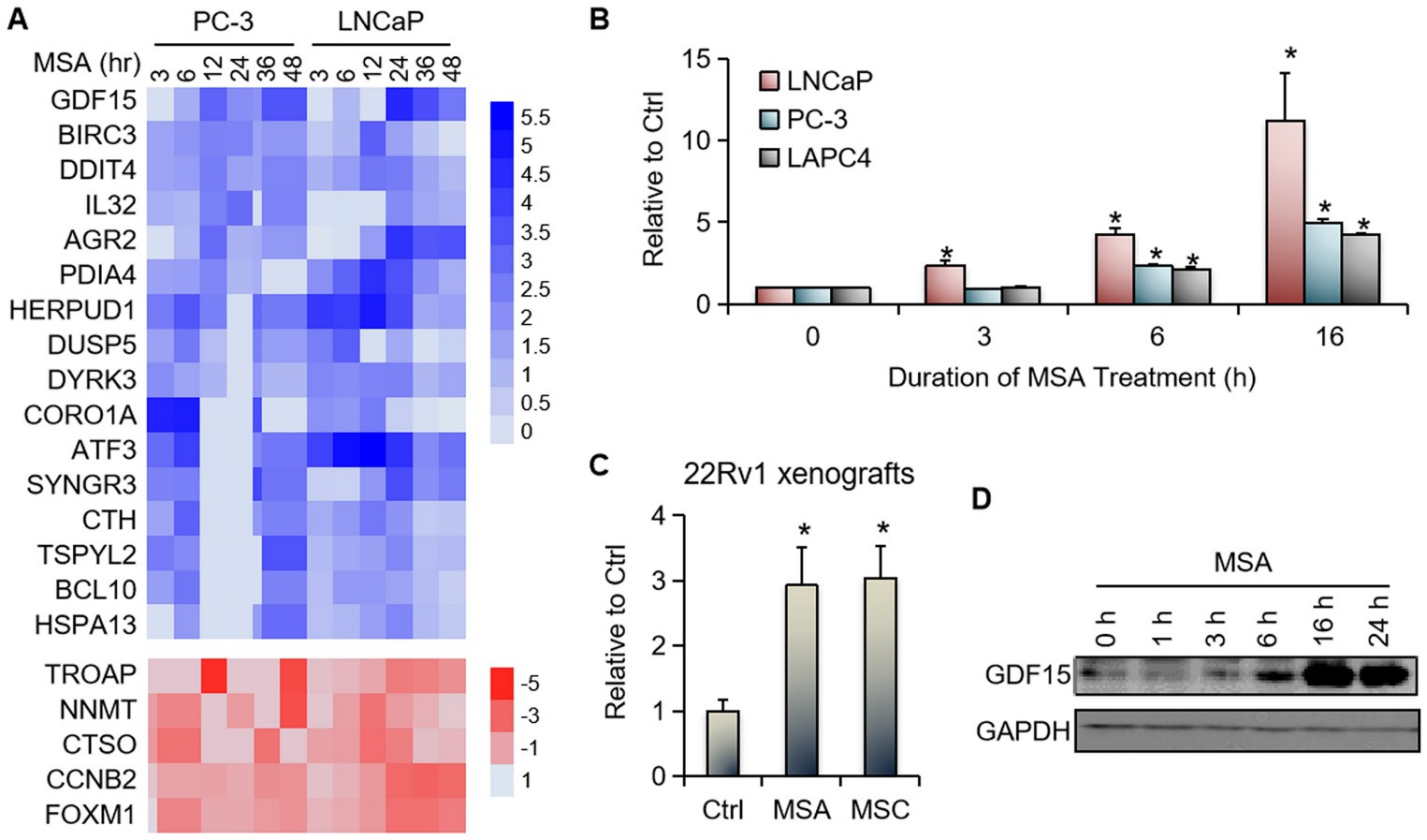
MSA upregulates the expression of GDF15. **A**. Microarray data showing GDF15 as one of the most highly induced genes by MSA in both PC-3 and LNCaP cells. Shown are all the genes that are modulated by MSA by at least a mean of 2 fold in both cell models. Colors indicate log2-transformed fold of change after MSA treatment. **B**. qRT-PCR analysis confirming MSA induction of GDF15 mRNA level in cultured cells. **C**. qRT-PCR showing induction of GDF15 mRNA level by MSA and MSC in 22Rv1 xenograft tumors (n = 3 for the control group; n = 4 for the MSA and MSC groups). **D**. Western blotting showing MSA induction of GDF15 protein level in LNCaP cells. All MSA treatment of cultured cells is at 10 μM. Bars, SEM. *, *P* < 0.05 from control using Student’s t test.

To identify key targets of MSA in prostate cancer, we analyzed the microarray data from the previous studies in PC-3 and LNCaP human prostate cancer cells treated with or without 10 μM MSA for 3, 6, 12, 24, 36, or 48 hours [8, 21]. Twenty-one genes (16 upregulated and 5 downregulated) were modulated by MSA by an average of more than 2 fold in both cell models, and GDF15 was among the most induced genes (Fig 1A). We then conducted qRT-PCR analysis to verify the microarray finding. In concordance with the pattern of changes observed in the microarray analysis, GDF15 mRNA was induced early in response to MSA treatment (Fig 1B and 1C). A significant increase was already evident at the 3-hour time point in LNCaP cells and at the 6-hour time point in PC-3 and LAPC-4 cells (Fig 1B). A similar induction was observed *in vivo* in 22Rv1 human prostate cancer xenograft tumors (Fig 1C). Moreover, MSC, which generates the same active metabolite as MSA in animals [27], exerted the same effect on GDF15 expression in 22Rv1 tumors (Fig 1C). The induction of GDF15 also occurred at the protein level (Fig 1D). Together, the above data from different preclinical models of prostate cancer support GDF15 as one of the highly induced and immediate targets of MSA.

### GDF15 contributes to MSA inhibition of cell proliferation and induction of apoptosis

**Fig 2:**
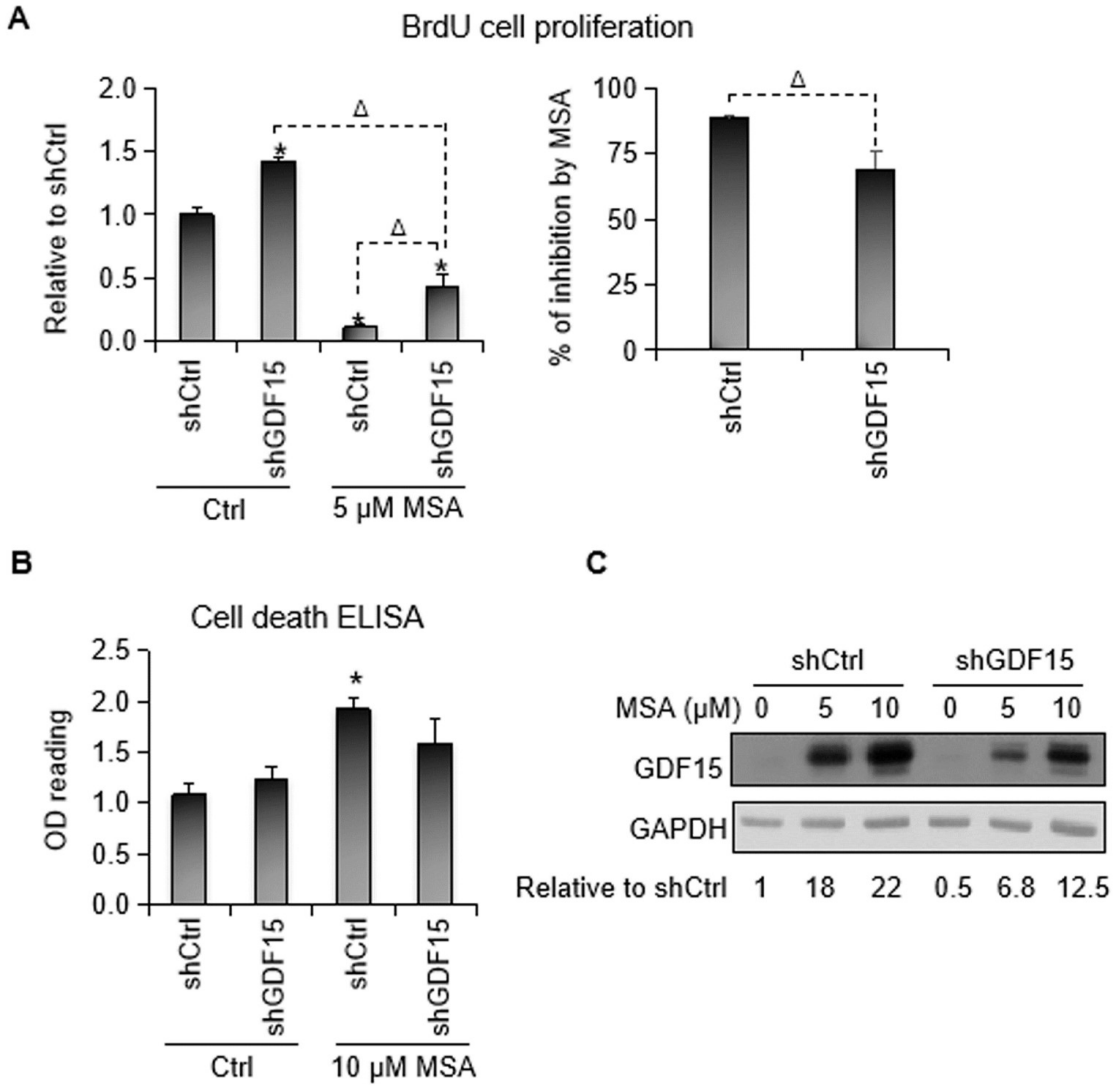
GDF15 knockdown attenuates MSA inhibition of cell proliferation and induction of apoptosis. LNCaP cells were transfected with the shGDF15 or the shCtrl construct. The cells were treated with MSA at 24 h after transfection. BrdU ELISA, Cell Death ELISA, and Western blot analysis were conducted at 24 h post-MSA treatment. **A**. BrdU cell proliferation ELISA showing GDF15 knockdown promoting cell proliferation and attenuating the inhibitory effect of MSA on cell proliferation in LNCaP cells and attenuating the inhibitory effect of MSA on cell proliferation in LNCaP cells. Data are presented as relative to the untreated shCtrl group (left panel) or percentage of inhibition by MSA in control and GDF15 knockdown cells (right panel). Δ, *P* < 0.05 using Student’s t test. **B**. Cell death ELISA showing GDF15 attenuating MSA induction of apoptosis in LNCaP cells. Data are presented as mean OD reading at 405 nm subtracted by the OD reading of incubation buffer for individual groups. **C**. Western blotting showing the efficacy of GDF15 knockdown. The numbers below the blots denote relative normalized intensities of the GDF15 protein bands compared to the untreated shCtrl value of 1. Treatment duration, 24 h. Bars, SEM. *, *P* < 0.05 from control using Student’s t test.

To investigate the functional significance of GDF15 induction by MSA, we knocked down the expression of GDF15 in LNCaP cells and assessed the impact on MSA inhibition of cell proliferation and induction of apoptosis. We lowered the dose of MSA from 10 μM to 5 μM for the BrdU ELISA assay in order to gauge the effect of MSA on cell proliferation in the absence of marked apoptosis. The BrdU ELISA data are presented as relative to the untreated shCtrl group or percentage of inhibition by MSA in shCtrl and shGDF15 cells (Fig 2A), and the apoptosis ELISA data are presented as mean OD reading at 405 nm subtracted by the OD reading of incubation buffer for individual groups (Fig 2B). MSA became less effective in inhibiting cell proliferation and inducing apoptosis after GDF15 knockdown, supporting an important role of GDF15 upregulation in mediating these biological activities of MSA. Additionally, the GDF15 knockdown cells proliferated faster than the control cells in the absence of MSA (Fig 2A), indicating a growth suppressive function of GDF15 in these cells.

### GDF15 RNA and protein levels are higher in primary human prostate cancers compared to metastatic tissues

**Fig 3:**
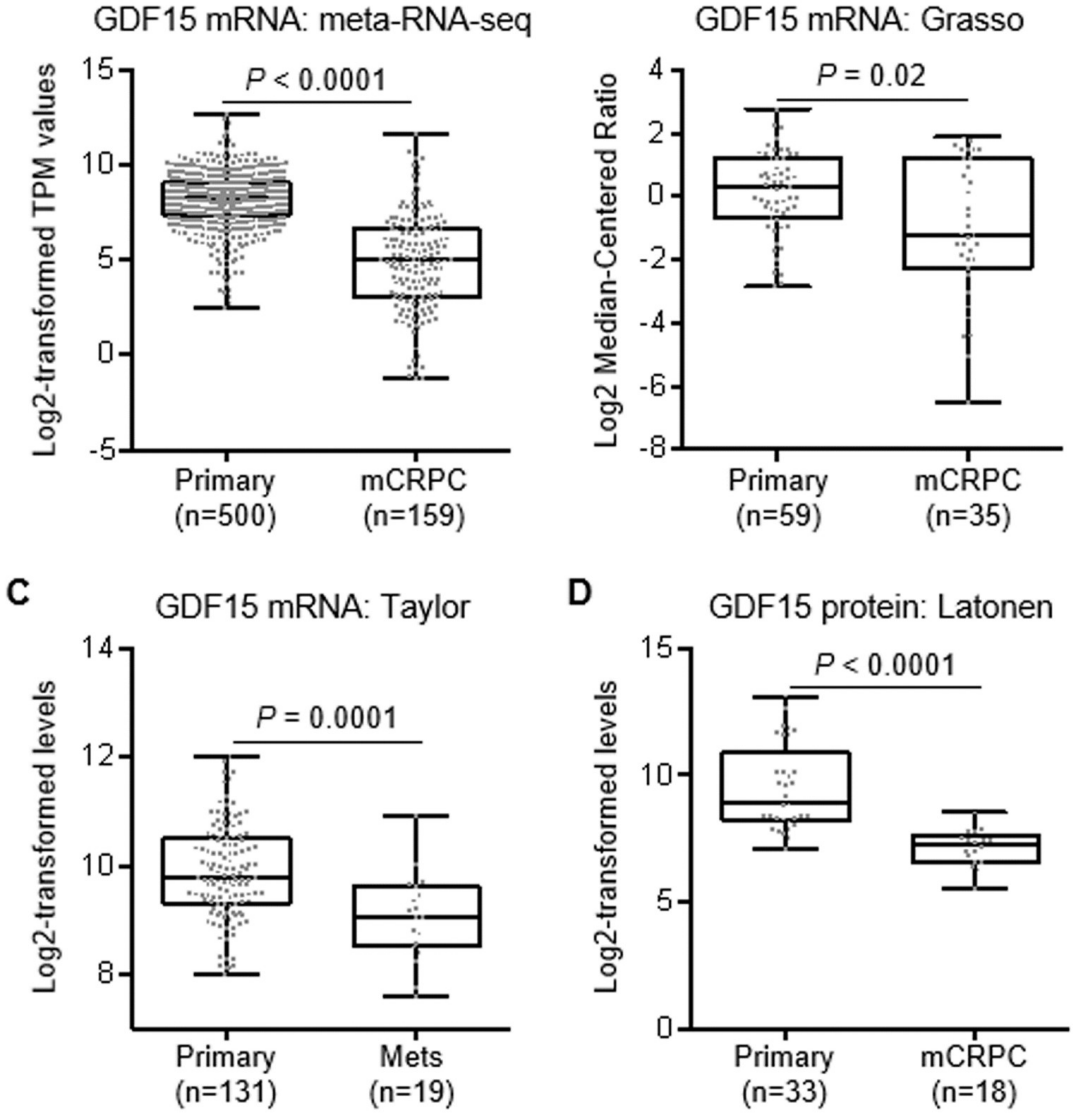
GDF15 RNA and protein levels are higher in primary human prostate cancer compared to metastatic samples. **A**, GDF15 RNA levels from RNA-seq data of 500 TCGA primary prostate cancer (primary) samples and 159 metastatic CRPC (mCRPC) samples from the SU2C, PROMOTE, and Beltran cohorts. **B**, GDF15 RNA levels from the Grasso microarray dataset (GSE35988) including 59 primary prostate cancer and 35 mCRPC samples. **C**, GDF15 RNA levels from the Taylor microarray dataset (GSE21034) including 131 primary and 19 metastatic prostate cancer samples. **D**, GDF15 protein levels from the Latonen proteomics dataset (PASS01126) including 33 primary prostate cancer and 18 mCRPC samples. TPM, transcripts per million. Numbers in parentheses, number of samples in each group. *P* values are from Mann-Whitney test.

To assess the clinical relevance of increased GDF15 expression in prostate cancer, we examined a number of published cohorts for GDF15 expression. Collating the RNA-seq data from 500 primary tumors in TCGA cohort and the RNA-seq data of 159 metastatic CRPC samples from the SU2C project (n = 51) [32], the PROMOTE study (n = 74) [33], and the Beltran cohort (n = 34) [34] showed significantly higher levels of GDF15 mRNA in primary prostate cancers compared to metastatic CRPC samples (Fig 3A). Analysis of the Grasso and the Taylor microarray datasets [36, 37] yielded the same result (Fig 3B and 3C). We further evaluated GDF15 protein levels in the 33 primary prostate cancer specimens and 18 metastatic CRPC samples included in the proteomics study by Latonen et al. [40] and found that GDF15 protein expression is also significantly higher in primary prostate cancers than in metastatic CRPC samples (Fig 3D). These results indicate that elevated levels of GDF15 are associated with less advanced prostate cancer.

### High GDF15 levels in primary prostate cancers are associated with better prognosis

**Fig 4:**
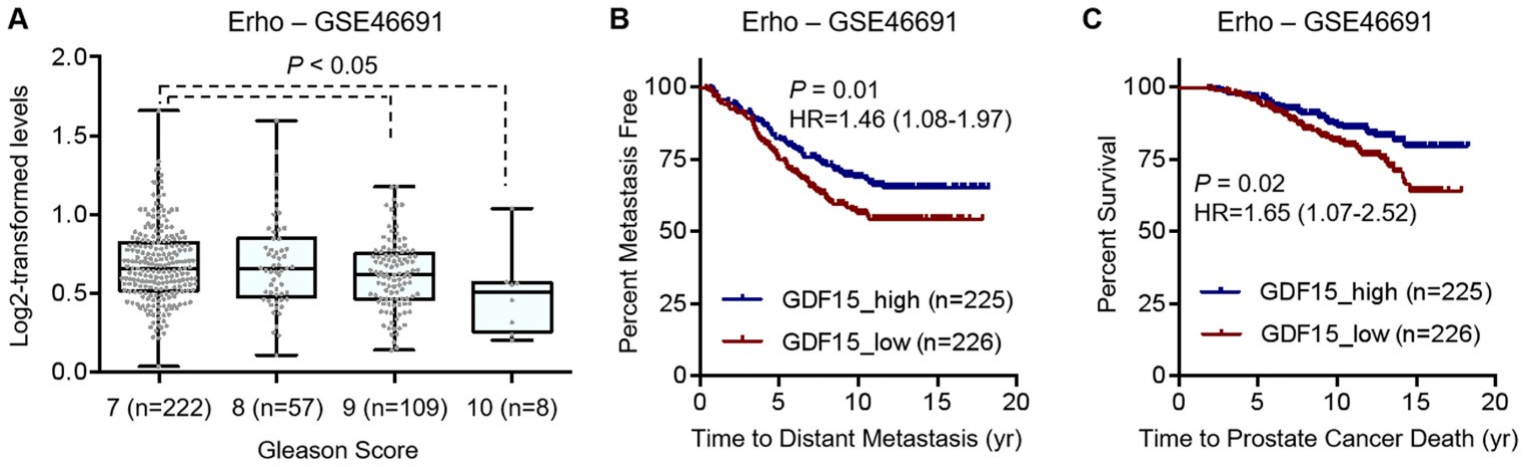
Low GDF15 levels are associated with high Gleason scores in primary tumors and shorter survival of the patients. Data are from the Erho GenomeDx Biosciences cohort. **A**, association of low GDF15 expression with high Gleason scores. *P* values are from Mann-Whitney test. **B & C**, Kaplan-Meier plots showing association of low GDF15 expression with shorter time to distant metastasis **(B)** and to prostate cancer-specific death **(C)** after radical prostatectomy. *P* values and hazard ratios (HR) are from Log-rank test. Numbers in parentheses, number of samples in each group.

To assess the prognostic value of GDF15 expression in primary prostate cancer, we analyzed the Erho microarray dataset, which contains 545 radical prostatectomy samples with a median follow-up of 16.9 years [38], for association with clinicopathologic parameters. As shown in Fig 4A, GDF15 mRNA level was lower in Gleason score 9 and 10 tumors than in Gleason score 7 tumors. Moreover, lower GDF15 levels were associated with shorter time to distant metastasis (Fig 4B) and to prostate cancer-specific death (Fig 4C). Together, the association between lower GDF15 expression and worse clinical outcome supports a role of GDF15 downregulation in prostate cancer progression.

### Same pathways are shared by GDF15 and MSA

**Fig 5:**
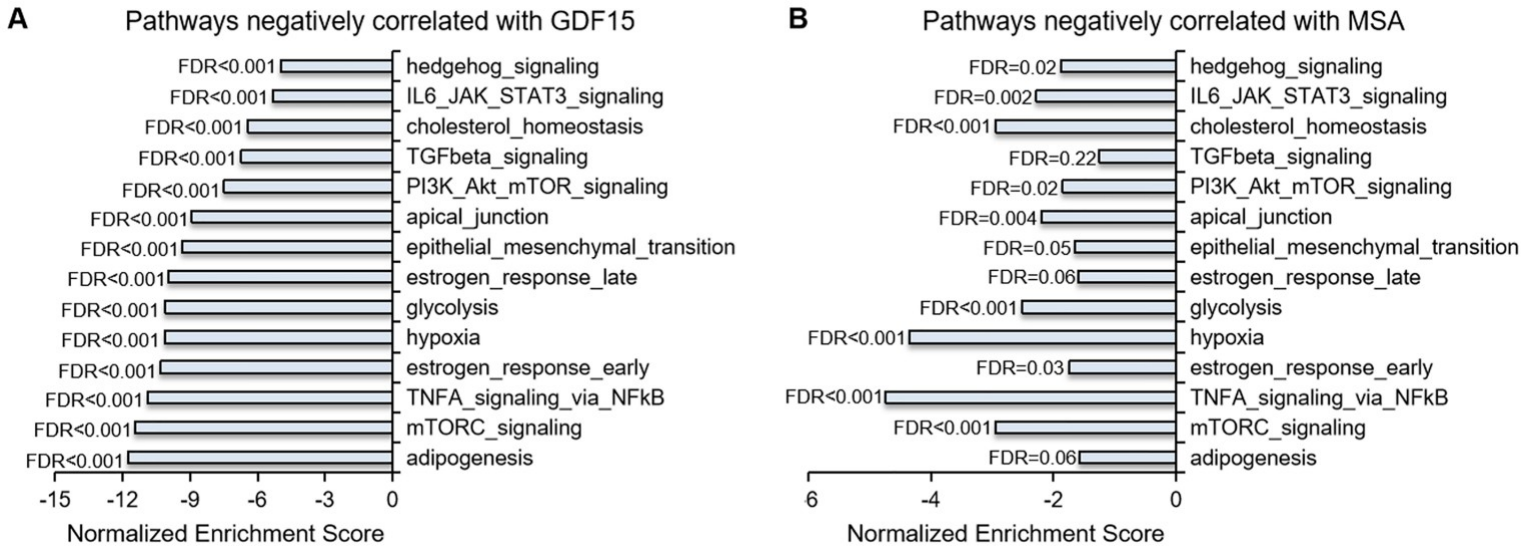
Same pathways are affected GDF15 and MSA. GSEA was conducted on the combined RNA-seq data of 500 TCGA primary prostate cancer (primary) samples and 159 metastatic CRPC (mCRPC) samples from the SU2C, PROMOTE, and Beltran cohorts and the microarray data from PC-3 cells treated with or without MSA. The same pathways that are negatively correlated with GDF15 levels in the combined RNA-seq data **(A)** are negatively correlated with MSA treatment in the PC-3-MSA microarray dataset **(B)**. FDR < 0.25 is considered significant.

To explore the mechanism by which GDF15 contributes to the antiproliferative and apoptosis-inducing activities of MSA, we conducted GSEA on the microarray data from PC-3 cells treated with or without MSA for different durations and the collated RNA-seq data of 500 primary tumors and 159 metastatic CRPC samples. We found that the pathways that were highly enriched in the transcriptional profiles of tumors that express a low level of GDF15 were similarly enriched in untreated control PC-3 cells compared to MSA-treated cells (Fig 5). Since most, if not all, of these pathways have been implicated in prostate cancer progression, suppressing the activities of these pathways by inducing GDF15 is consistent with the antitumor effect of MSA in prostate cancer.

## Discussion

Since the first report of the growth inhibitory activities of MSA in prostate cancer in 2001 [11], numerous studies have been conducted to unravel the underlying mechanisms. However, our understanding of the immediate targets that are key to the growth inhibitory effects of MSA is still limited. Here, we showed that GDF15 is one of the most highly induced genes by MSA in multiple preclinical prostate cancer models *in vitro* and *in vivo* and that the induction occurred rapidly. GDF15 has been shown to function either in the nucleus or as a secreted protein [30, 42]. Nuclear GDF15 was shown to inhibit TGF-β1-induced Smad signaling to mitigate cell migration and invasion [42]. Consistently, we found that TGF-β signaling is negatively correlated with GDF15 levels in clinical prostate cancer samples. Moreover, TGF-β signaling is also negatively correlated with MSA treatment in cultured cells. The data indicate that nuclear GDF15 may be involved in mediating the growth-suppressive effects of MSA through inhibiting TGF-β signaling. As for secreted GDF15, the only receptor that has been identified to date is the orphan receptor glial-derived neurotrophic factor-family receptor α-like (GFRAL), which was reported to mediate the metabolic effects of GDF15 [43]. However, through surveying our collated RNA-seq data, we found that the levels of GFRAL mRNA in benign prostate tissues, primary prostate cancer, and metastatic CRPC samples are very low, with mean TPM values of 0.22, 0.13, and 0.13, respectively, as opposed to 134, 459, and 127, respectively, for GDF15 (S3 Fig). Thus, secreted GDF15 is likely to signal through a different receptor in the prostate.

Several transcription factors have been indicated to regulate the expression of GDF15 [44]. Of particular interest are p53 [45, 46] and androgen receptor (AR) [47] as they are also immediate targets of MSA [6, 7, 22, 48]. However, since MSA is able to induce GDF15 expression in both p53 wild-type and p53-null cells and in both AR-expressing and AR-null cells, GDF15 upregulation by MSA is likely to be independent of p53 and AR. Endoplasmic reticulum (ER) stress has also been shown to increase GDF15 expression [49], and MSA is known to induce ER stress in prostate cancer cells [19]. Nonetheless, when we overexpressed GRP78, the rheostat of ER stress transducers and the overexpression of which mitigates MSA induction of ER stress [19], in LNCaP cells, the effect on MSA upregulation of GDF15 was minimal (S4 Fig), excluding ER stress as an underlying mechanism. Understanding how MSA induces GDF15 expression is an area of our ongoing research.

Both tumor-suppressing and tumor-promoting functions of GDF15 in the prostate have been implicated [30]. For example, immunohistochemical staining of prostatectomy specimens showed an inverse association between GDF-15 levels and prostatic inflammation, a known prostate tumor-promoting factor [50]. TRAMP mice bearing a germline deletion of GDF15 develop larger prostatic tumors than TRAMP mice with wild-type GDF15 [51]. On the other hand, both tissue and serum levels of GDF15 have been shown to be elevated in prostate cancer patients relative to non-cancerous individuals [52–54]. Ectopic expression of GDF15 has been shown to decrease cell growth and induce apoptosis in some prostate cancer cell models [55, 56] but increase cell growth and metastatic potential in some other models or other conditions [57–59].

The seemingly paradoxical role of GDF-15 in prostate cancer might be attributed to the biphasic regulation of GDF15 expression in early-stage of tumor development versus during tumor progression. Albeit GDF15 expression is downregulated in metastatic CRPCs compared to primary tumors, we found that the levels of GDF15 RNA and protein are higher in primary prostate tumors than in benign prostate tissues (S1 and S2 Figs). This is consistent with the report from a previous study, in which immunohistochemical staining of tissue microarrays containing over 1600 cores of benign prostate tissues, low- and high-grade prostatic intraepithelial neoplasia, and primary prostate cancer samples showed a progressive increase of GDF15 protein levels from benign tissues to low- and high-grade prostatic intraepithelial neoplasia and to prostate cancer [60]. However, the same study showed that in prostate cancer samples, lower GDF15 expression is associated with higher Gleason pattern and pathologic stage and increased risk of relapse after surgery [60]. This biphasic regulation of GDF15 expression was also observed in another study, which demonstrated a higher level of GDF15 mRNA in prostate cancer than in benign prostate tissues but a lower level of GDF15 mRNA in moderately and poorly differentiated adenocarcinomas than in well-differentiated prostate cancer [61]. Therefore, the role of GDF15 in prostate cancer is likely to be stage specific. It may promote early stages of tumorigenesis but suppress the progression of advanced prostate cancer. Consequently, the role of GDF15 in mediating the growth-suppressive effects of MSA is likely to be specific to prostate cancer cells that are at an advanced stage.

In summary, the work described herein demonstrates that GDF15 is an immediate target of MSA in prostate cancer cells and that GDF15 induction contributes to MSA inhibition of cell proliferation and induction of apoptosis. Since low GDF15 expression is associated with more aggressive prostate cancer and worse clinical outcome, induction of GDF15 may be a viable approach to treat advanced prostate cancer and prevent prostate cancer progression. By improving our understanding of the mechanisms of action of MSA in prostate cancer, these findings will help future design of clinical trials using MSA to treat prostate cancer.

## Supporting information

S1 Fig GDF15 mRNA levels are higher in primary prostate cancer compared to benign prostate tissues. **A**, Box and whisker (min to max) plots of GDF15 mRNA levels from RNA-seq data of 51 TCGA benign prostate tissues, 500 TCGA primary prostate cancer (primary) samples, and 159 metastatic CRPC (mCRPC) samples from the SU2C, PROMOTE, and Beltran cohorts. **B**, Box and whisker (min to max) plots of GDF15 RNA levels from the Grasso microarray dataset (GSE35988). TPM, transcripts per million. Numbers in parentheses, number of samples in each group. P values are from Mann-Whitney test.
(TIF)

S2 Fig GDF15 protein levels are higher in primary human prostate cancer compared to benign prostate tissues. The data are from the Latonen proteomics dataset (PASS01126) including 33 primary prostate cancer specimens and 18 metastatic CRPC samples. Numbers in parentheses, number of samples in each group. *P* values are from Mann-Whitney test.
(TIF)

S3 Fig GFRAL mRNA levels are low in the prostate. **A & B**, GFRAL and GDF15 mRNA levels from RNA-seq data of 51 TCGA benign prostate tissues, 500 TCGA primary prostate cancer (primary) samples, and 159 metastatic CRPC (mCRPC) samples from the SU2C, PROMOTE, and Beltran cohorts. TPM, transcripts per million. Numbers in parentheses, number of samples in each group. Bars, SEM with 95% confidence interval.
(TIF)

S4 Fig GRP78 knockdown does not affect MSA upregulation of GDF15. LNCaP cells were transfected with the shGRP78 or the shCtrl construct. The cells were treated with 10 μM MSA at 24 h after transfection and harvested at 3 or16 h after treatment for qRT-PCR analysis of GDF15 mRNA levels. *, *P* < 0.05 from the respective control using Student’s t test.
(TIF)
