# Supplementary figures and images for: Role of GDF15 in methylseleninic acid-mediated inhibition of cell proliferation and induction of apoptosis in prostate cancer cells
Source: PLoS One. 2019 Sep 20;14(9):e0222812. doi: 10.1371/journal.pone.0222812 (PMC6754141; doi:10.1371/journal.pone.0222812)

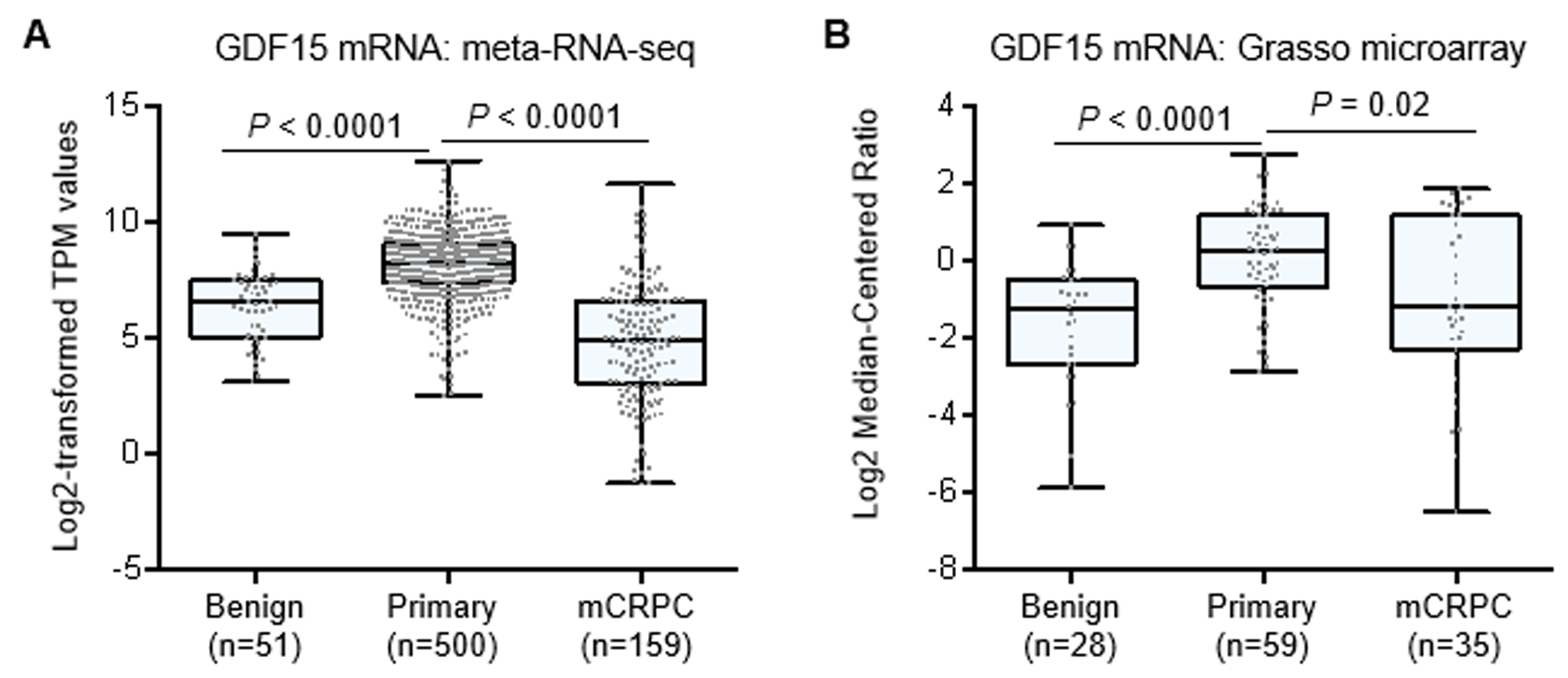

Supplement: S1 Fig — A, Box and whisker (min to max) plots of GDF15 mRNA levels from RNA-seq data of 51 TCGA benign prostate tissues, 500 TCGA primary prostate cancer (primary) samples, and 159 metastatic CRPC (mCRPC) samples from the SU2C, PROMOTE, and Beltran cohorts. B, Box and whisker (min to max) plots of GDF15 RNA levels from the Grasso microarray dataset (GSE35988). TPM, transcripts per million. Numbers in parentheses, number of samples in each group. P values are from Mann-Whitney test. (TIF) [file pone.0222812.s001.tif]

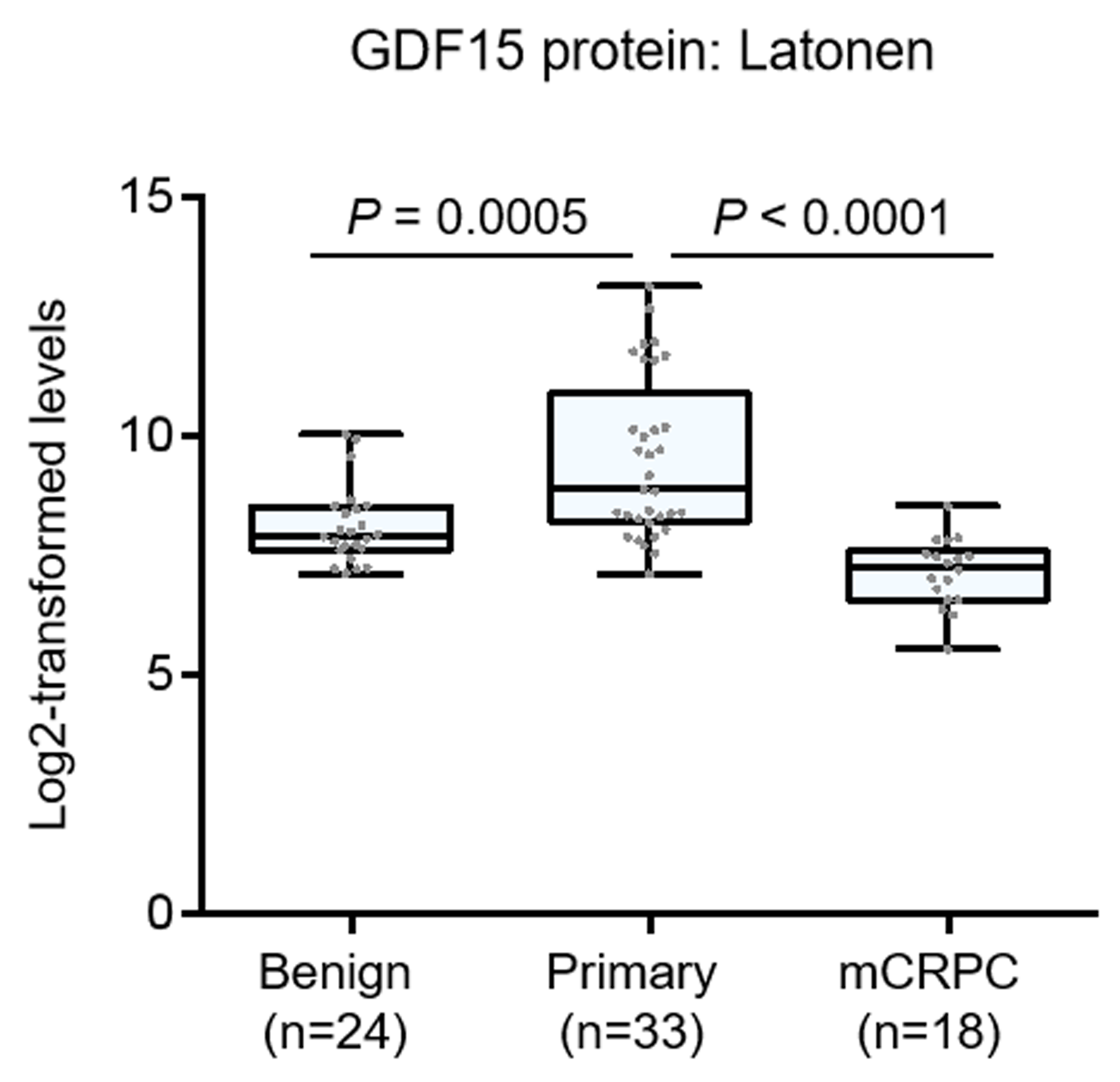

Supplement: S2 Fig — The data are from the Latonen proteomics dataset (PASS01126) including 33 primary prostate cancer specimens and 18 metastatic CRPC samples. Numbers in parentheses, number of samples in each group. P values are from Mann-Whitney test. (TIF) [file pone.0222812.s002.tif]

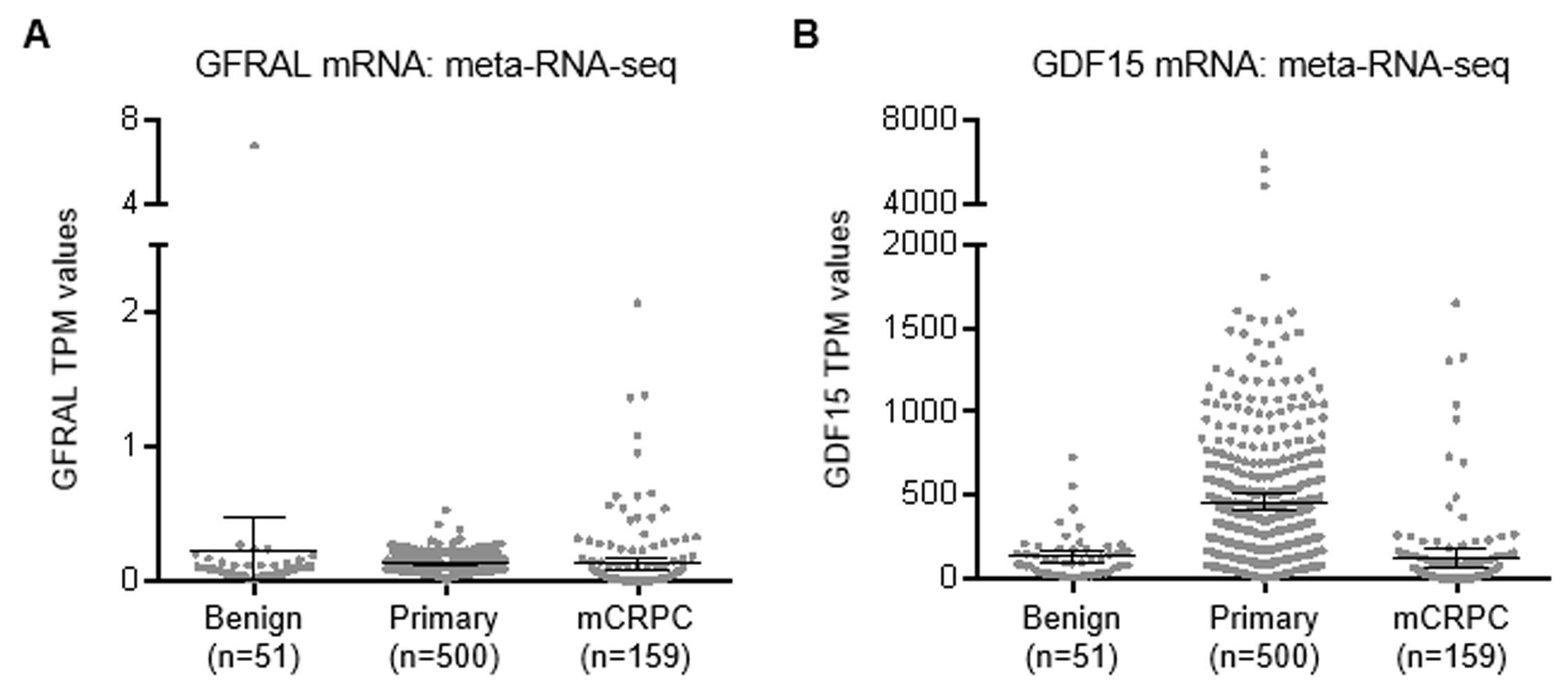

Supplement: S3 Fig — A & B, GFRAL and GDF15 mRNA levels from RNA-seq data of 51 TCGA benign prostate tissues, 500 TCGA primary prostate cancer (primary) samples, and 159 metastatic CRPC (mCRPC) samples from the SU2C, PROMOTE, and Beltran cohorts. TPM, transcripts per million. Numbers in parentheses, number of samples in each group. Bars, SEM with 95% confidence interval. (TIF) [file pone.0222812.s003.tif]

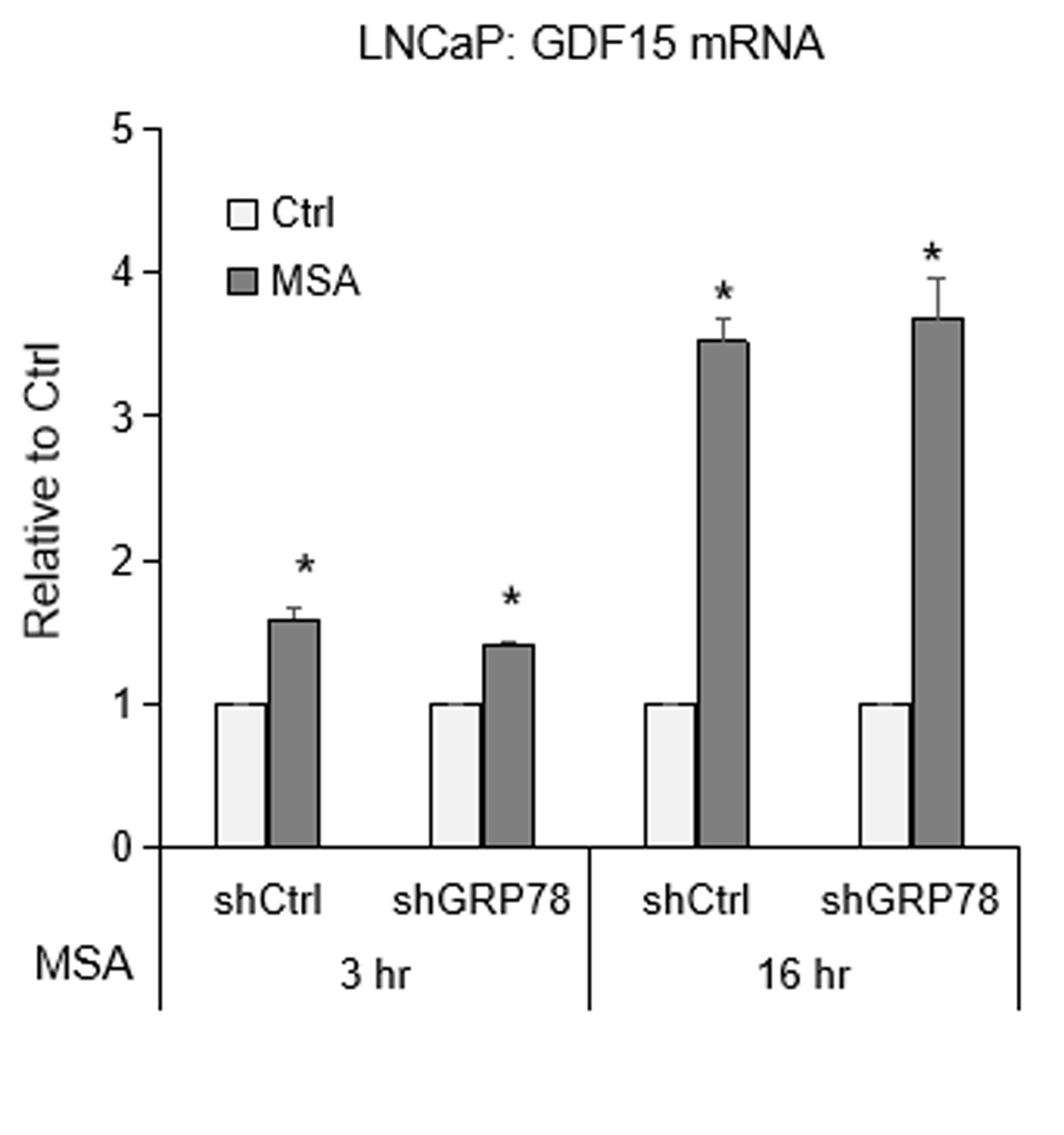

Supplement: S4 Fig — LNCaP cells were transfected with the shGRP78 or the shCtrl construct. The cells were treated with 10 μM MSA at 24 h after transfection and harvested at 3 or16 h after treatment for qRT-PCR analysis of GDF15 mRNA levels. *, P < 0.05 from the respective control using Student’s t test. (TIF) [file pone.0222812.s004.tif]
